# Diagnosis of atlanto-occipital dissociation: Standardised measurements of normal craniocervical relationship in finless porpoises (genus *Neophocaena*) using postmortem computed tomography

**DOI:** 10.1038/s41598-018-26866-8

**Published:** 2018-05-31

**Authors:** Brian C. W. Kot, Derek K. P. Chan, Adams H. L. Yuen, Henry C. L. Tsui

**Affiliations:** 1 School of Medical and Health Sciences, Tung Wah College, Homantin, Kowloon, Hong Kong SAR, China; 2 Department of Applied Biology and Chemical Technology, The Hong Kong Polytechnic University, Hunghom, Kowloon, Hong Kong SAR, China; 3 Office of the Vice-President (Development and External Relations), City University of Hong Kong, Kowloon, Hong Kong SAR, China

## Abstract

Due to the different craniocervical structures in humans and cetaceans, a standardised method assessing the normal craniocervical relationship in cetaceans is lacking, causing difficulties in defining the presence of atlanto-occipital dissociation (AOD) in cetaceans. The present study aimed to 1) describe a novel standardised method of determining the normal craniocervical relationships, and 2) define the 95% accuracy range of the normal craniocervical relationship in finless porpoises (genus *Neophocaena*), that allowed AOD diagnosis. Fifty-five out 83 stranded or by-caught finless porpoise carcasses were analyzed in term of their craniocervical relationship in dorsal-ventral and medial-lateral dimension, using postmortem computed tomography measurements. The normal craniocervical relationship in both dorsal-ventral (mean BD/OV: 0.87 ± 0.24 [2 *SD*]) and medial-lateral dimension (mean VR/VL: 0.98 ± 0.17 [2 *SD*]) was first defined. The 95% accuracy ranges of the normal craniocervical relationship in dorsal-ventral (0.63–1.11) and medial-lateral dimension (0.82–1.15) were proposed. The baseline ranges could facilitate AOD assessment, and provide an objective means of record for AOD related injury and death of cetaceans caused by anthropogenic trauma. The technique developed may be applied to live cetaceans with abnormal craniocervical relationship to aid diagnosis and guide corrective therapy.

## Introduction

The cetacean neck is foreshortened and the cervical vertebrae fused in different degrees^1,2^. Such specialized anatomy hinders twisting or turning of the cervical spine below C2, which in turn increases rigidity and stability of the large cranium and mandible^3^. The neck muscles and ventral thorax contribute to flexion and rotation of the head, while extension of the neck is limited by the fusion of the cervical vertebrae and their relation to the cranium and thorax. Splenius capitis muscles connect the cervical spine to the atlas (C1) and the back of the cranium, either directly or through other ventral neck muscles, resulting restriction of mobility within the neck region^3^. These strong neck muscles, together with a network of corresponding ligamentous attachments, provide stability to the atlanto-occipital joint (AOJ). The AOJ is functionally important for hearing and echolocation^4–6^. It controls all movements of the cetacean head with respect to the spine, allowing rotation of the head around a transverse axis through the 2 occipital condyles. Injury to the craniocervical structures may have devastating impacts on cetaceans.

Atlanto-occipital dissociation (AOD), a severe form of high-velocity, high-force AOJ injury includes both atlanto-occipital dislocation and atlanto-occipital subluxation. Various patterns of AOD have been reported in humans^7,8^, domestic^9,10^, and wild animals^11–13^, but rarely in marine mammals. Wild coastal cetaceans are believed to face risk of injury from by-catch and heavy marine traffic. The only published report described an AOD with partial severance of the spinal cord in a common dolphin washed ashore, together with a mass of monofilament gill net around its tail stock^14^. Vessel collision has been extensively reported in small^15–17^ and large cetaceans^18–20^. Obvious traumatic wounds like deep propeller scars and severed spines, tail flukes and fins, have been recorded in both live and stranded animals that had been victims of vessel collisions. Blunt trauma can also result from vessel collisions, leading to major internal injury and bleeding, in which may include AOD^21^.

Diagnostic imaging studies play a pivotal role in the diagnosis and management of AOD patients. Direct measurement of craniocervical relationships on plain lateral cervical spinal radiographs is recognized as a conventional radiological assessment of AOD in humans^22^. Suffering from AOD may be overtly obvious on plain radiographs with severe dislocation, but these injuries are usually fatal. In survivors, the radiographic changes are often subtle, making diagnosis more challenging. In recent years, computed tomography (CT) has become the standard radiologic technique for diagnosis of fractures and dislocations in the craniocervical region. Postmortem computed tomography (PMCT) can be a valuable tool for identification and documentation of osteological structures and the adjacent soft tissues that are not preserved in osteological collections. Spatial relationship between soft tissues and bones at AOJ can be selectively investigated *in situ* in their true dimensions and positions. The PMCT of AOD is first evaluated by Madadin and his team^23^, suggested that standardised measurements in assessing AOJ was applicable in human cadavers, with no effects of postmortem changes on the measurement of basion-axial interval as relied upon clinically.

In the course of the pioneering virtopsy-driven stranding response program in Hong Kong and adjacent waters, virtopsy using PMCT and postmortem magnetic resonance imaging (PMMRI) have been implemented to provide supplementary or complementary information to conventional necropsy^24,25^. Kot and his team suspected a high incidence of abnormal diastasis of AOJ in stranded cetaceans using PMCT^26^. To the best of our knowledge, the formal literature is devoid of any reference to standardise radiographic measurements for AOD diagnosis in cetaceans. The present study aimed to 1) describe a novel standardised method of determining the normal craniocervical relationships, and 2) define the 95% accuracy range of the normal craniocervical relationship in finless porpoises (genus *Neophocaena*), as obtained on reconstructed PMCT images for the diagnosis of AOD.

## Results

Craniocervical relationship of 55 finless porpoise carcasses (10 *Neophocaena phocaenoides* [NP]: 4 males, 5 females, 1 undetermined sex; 39 *Neophocaena asiaeorientalis sunameri* [NAS]: 22 males, 16 females, 1 undetermined sex; 6 *Neophocaena asiaeorientalis asiaeorientalis* [NAA]: 3 males, 3 females) with intact cranium and atlas were analyzed.

**Figure 1:**
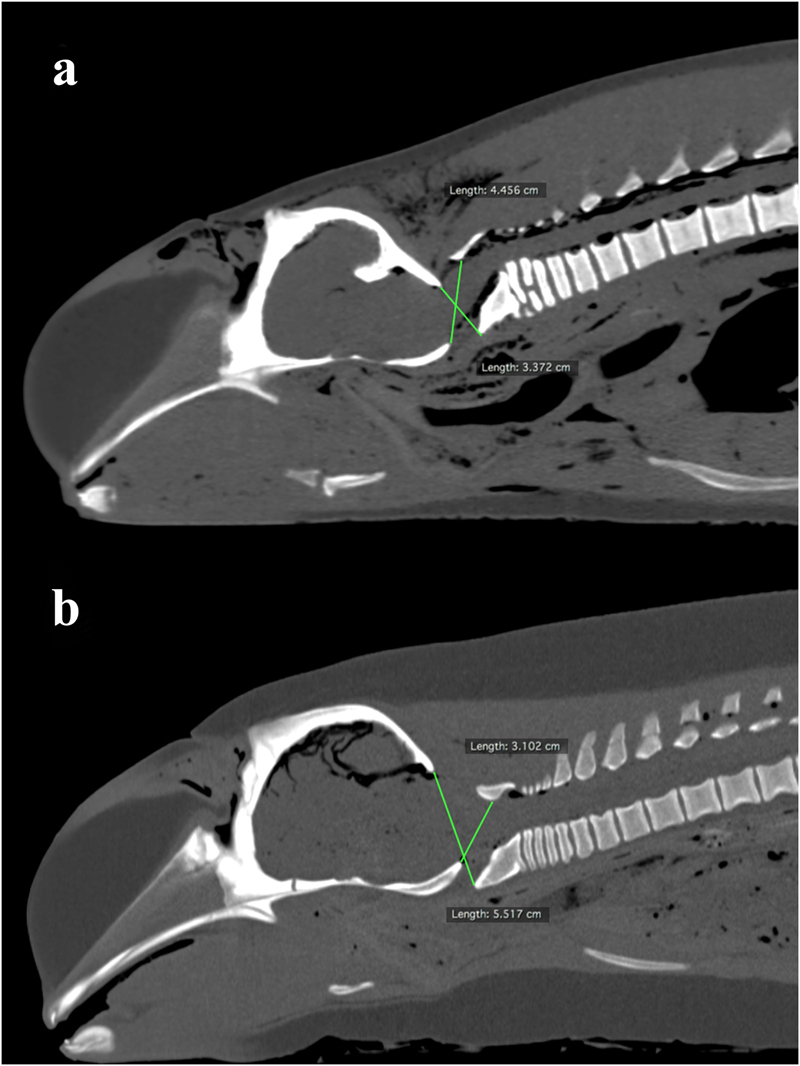
Reconstructed midsagittal postmortem computed tomography images of finless porpoise diagnosed with atlanto-occipital dissociation (AOD) in dorsal-ventral dimension, (**a**) dorsally displaced AOD and (**b**) ventrally displaced AOD.

**Figure 2:**
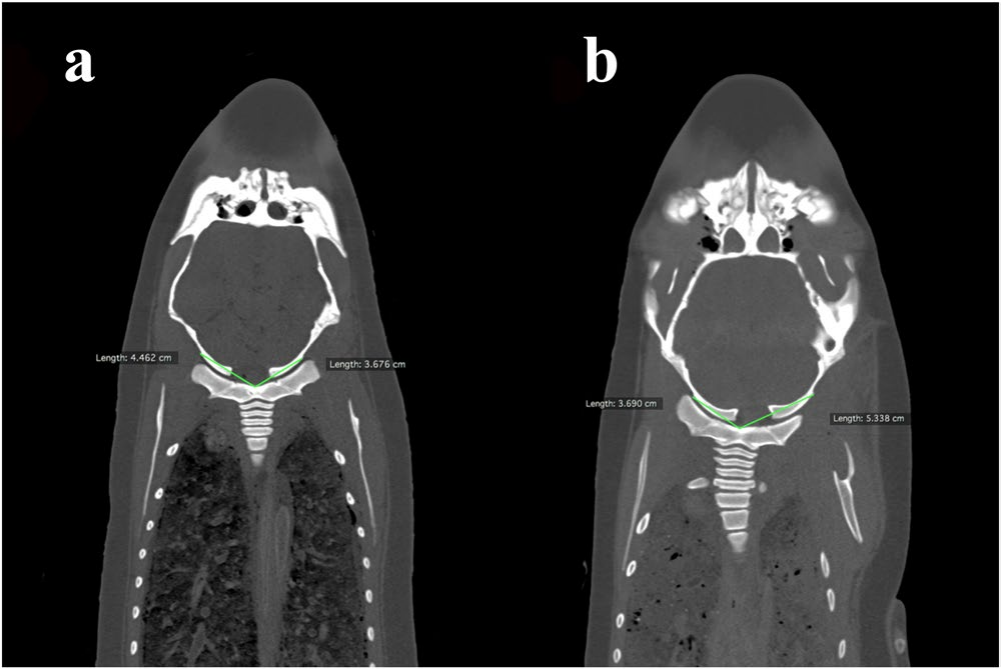
Reconstructed coronal postmortem computed tomography images of finless porpoise diagnosed with atlanto-occipital dissociation (AOD) in medial-lateral dimension, (**a**) right laterally displaced AOD and (**b**) left laterally displaced AOD.

Twenty-eight out of 55 carcasses (51%) demonstrated a normal craniocervical relationship. Twenty-seven out of 55 carcasses (49%) were diagnosed with AOD in dorsal-ventral dimension (Fig. 1) and medial-lateral dimension (Fig. 2). Two finless porpoises were diagnosed with AOD in dorsal-ventral and medial-lateral dimension concurrently (one exhibited left laterally displaced AOD [LAOD] and ventrally displaced AOD [VAOD]; one exhibited LAOD and dorsally displaced AOD [DAOD]). Twenty-four (89%) and 3 (11%) out of 27 carcasses (11%) were identified as postmortem and antemortem AOD respectively. Atlanto-occipital dissociation could be considered as the cause of death in the 3 antemortem AOD carcasses since no other significant gross pathology was noted and presence of whole or partially digested prey in stomach in the conventional necropsy.

The mean ratio between basion-dorsal arch of atlas (BD) and opisthion-ventral arch of atlas (OV) of carcasses (non-AOD exhibited group) demonstrated that a normal craniocervical relationship in dorsal-ventral dimension was 0.87 ± 0.24 (2 standard deviation [*SD*]) (*n* = 43; ranged 0.71–1.14) (Table 1). The BD/OV of carcasses diagnosed with DAOD (mean BD/OV = 0.54 ± 0.10 [2 *SD*]; *n* = 7; ranged 0.48–0.61) was significantly (*P* < 0.05) smaller than that of non-AOD exhibited group. The BD/OV of carcasses diagnosed with VAOD (mean BD/OV = 1.39 ± 0.19 [2 *SD*]; *n* = 5; ranged 1.26–1.50) was significantly (*P* < 0.05) greater than that of non-AOD exhibited group. The 95% accuracy range of BD/OV was 0.63 to 1.11. A finless porpoise carcass with BD/OV measured less than 0.63 or greater than 1.11 might anticipate to exhibit DAOD or VAOD respectively.

**Table 1:** Comparison of the mean ratio between basion-dorsal arch (pars basilaris-arcus dorsalis) of atlas (BD) and opisthion-ventral arch (squama occipitalis-arcus ventralis) of atlas (OV) between carcasses with and without sign of atlanto-occipital dissociation (AOD) in dorsal-ventral dimension, including the significance level among carcasses in non-AOD exhibited group and dorsally displaced AOD (DAOD)/ventrally displaced AOD (VAOD) exhibited group, with the use of Kruskal–Wallis test with Dunn’s multiple comparison tests as post-hoc tests.

Group (*n*)	Mean of BD/OV ± 2 *SD*	Range of BD/OV
DAOD exhibited group (7)	0.54 ± 0.10	0.48–0.61
VAOD exhibited group (5)	1.39 ± 0.19	1.26–1.50
Non-AOD exhibited group (43)	0.87 ± 0.24	0.71–1.14
**Groups compared**	***P*** **value**	
DAOD exhibited group vs. Non-AOD exhibited group	*P* < 0.05	
VAOD exhibited group vs. Non-AOD exhibited group	*P* < 0.05	

The mean ratio between ventral tubercle of fused cervical vertebral body to left outer margin of occipital condyle (VL) and ventral tubercle of fused cervical vertebral body to right outer margin of occipital condyle (VR) of carcasses (non-AOD exhibited group) demonstrated that a normal craniocervical relationship in dorsal-ventral dimension was 0.98 ± 0.17 (2 *SD*) (*n* = 38; ranged 0.60–1.50) (Table 2). The VR/VL of carcasses diagnosed with right laterally displaced AOD (RAOD) (mean VR/VL = 0.70 ± 0.01 [2 *SD*]; *n* = 10; ranged 0.60–0.79) was significantly (*P* < 0.05) smaller than that of non-AOD exhibited group. The VR/VL of carcasses diagnosed with LAOD (mean VR/VL = 1.37 ± 0.16 [2 *SD*]; *n* = 7; ranged 1.29–1.50) was significantly (*P* < 0.05) greater than that of non-AOD exhibited group. The 95% accuracy range of VR/VL was 0.82 to 1.15. A finless porpoise carcass with VR/VL measured less than 0.82 or greater than 1.15 might anticipate to exhibit RAOD or LAOD respectively.

**Table 2:** Comparison of the mean ratio between ventral tubercle of fused cervical vertebral body to left outer margin of occipital condyle (VL) and ventral tubercle of fused cervical vertebral body to right outer margin of occipital condyle (VR) between carcasses with and without sign of atlanto-occipital dissociation (AOD) in medial-lateral dimension, including the significance level among carcasses in non-AOD exhibited group and left laterally displaced AOD (LAOD)/right laterally displaced AOD (RAOD) exhibited group, with the use of Kruskal–Wallis test with Dunn’s multiple comparison tests as post-hoc tests.

Group (*n*)	Mean of VR/VL ± 2 *SD*	Range of VR/VL
LAOD exhibited group (7)	1.37 ± 0.16	1.29–1.50
RAOD exhibited group (10)	0.70 ± 0.01	0.60–0.79
Non-AOD exhibited group (38)	0.98 ± 0.17	0.60–1.50
**Groups compared**	***P*** **value**	
LAOD exhibited group vs. Non-AOD exhibited group	*P* < 0.05	
RAOD exhibited group vs. Non-AOD exhibited group	*P* < 0.05	

## Discussion

In humans, conventional radiography has historically been the modality of choice to assess traumatic upper cervical spine injuries. However, Tepper *et al*.^27^ have suggested that approximately 50% of the craniocervical injuries were missed during the initial conventional radiographic evaluation. With advances in the cross-sectional imaging technology, a transition from conventional radiography to PMCT with multiplanar reconstructions for assessment of AOD has occurred. Assessment of the extent of atlanto-occipital diastasis can be conducted in PMCT, allowing a more accurate and faster observation on the dynamic nature of articular displacement compared to conventional necropsy. The shortcomings of performing measurements on radiographs do not apply to PMCT because bony landmarks are presented without superimposition^10^. To the best of our knowledge, the formal literature is devoid of any information regarding the standardised radiographic assessment of AOD in cetaceans. The present study was the first to describe a novel standardised method for the determination of normal craniocervical relationships in stranded finless porpoises using PMCT.

Given the complex craniocervical anatomical relationships and biomechanical factors involved in AOD, a single measurement from imaging studies may not be able to define AOD. Efforts have been paid to diagnose this often overlooked entity in humans, all methods seek to assess damage of the structures stabilizing the AOJ as a common goal^28^. In the present study, indication of craniocervical relationship in medial-lateral dimension was also first included in addition to that in the dorsal-ventral dimension. This was done in consideration of discrepancies in atlanto-occipital relationship between cetaceans and humans^1,2^ and possible predisposition of the animal’s body trunk to anthropogenic blunt force impact associated with vessel strikes^29–31^.

Regarding the craniocervical relationship in dorsal-ventral dimension, the data yielded significant differences (*P* < 0.05) in mean BD/OV between non-AOD exhibited group and DAOD exhibited group, and between non-AOD exhibited group and VAOD exhibited group. The 95% accuracy range of BD/OV in finless porpoises (0.63 to 1.11) was found to be similar to that of BC/OA in humans (0.59 to 0.95)^22^, which is considered as a normal craniocervical relationship in dorsal-ventral dimension. This 95% accuracy range of BD/OV may imply some degree of deviation in head dorsal-ventral movement in respect to the vertebral column, which perhaps facilitates echolocation and locomotion in cetaceans^3^. Mammalian echolocators perceive external environment through self-generated sound echoes, and respective head movements have been proven to affect the performance of echolocation and corrective identification of two-dimensional shape^32^, which may be critical in cetacean foraging strategies and social interaction^5,33,34^.

Regarding the craniocervical relationship in medial-lateral dimension, the data yielded a significant difference (*P* < 0.05) in mean VR/VL between non-AOD exhibited group and LAOD exhibited group, and between non-AOD exhibited group and RAOD exhibited group. The 95% accuracy range of VR/VL in finless porpoises was 0.82 to 1.15, which can be considered as a normal craniocervical relationship in medial-lateral dimension. The narrower 95% accuracy range of VR/VL (0.82 to 1.15), when compared to that of BD/OV (0.63 to 1.11), may suggest a limited lateral head movement in cetaceans. The union of cranium and atlas is unique in cetaceans - a short cervical tract, fused vertebrae in different degrees and hemispherical occipital condyles^3^. With this craniocervical anatomy, cetaceans are allowed to move and rotate along three different axes^1^, but not to twist or turn the cervical spine below axis (C2).

In the present study, similar frequencies of occurrence were found in all AOD exhibited groups (DAOD: *n* = 7; VAOD: *n* = 5; LAOD: *n* = 7; RAOD: *n* = 10). Half of the analyzed carcasses (27 out of 55) were diagnosed with AOD (antemortem AOD: *n* = 3; postmortem AOD: *n* = 24). Despite reports of the diagnosis and mechanism of AOD in cetaceans being scarce and lacking in detail^35^, we proposed the potential causes of AOD under 2 categories, i.e. anthropogenic events and natural origins. The former may include the association of violent struggling of entangled individual^14,31^, and hyperextension injury secondary to vessel collision^31^, while the latter may include epimeletic behavior^36,37^, and intra- or inter-specific aggression behavior^31^.

In this present study, PMCT measurements of the normal craniocervical relationship in finless porpoises were emphasized. The yielded baseline ranges could facilitate AOD assessment, and serve as an objective means of record for AOD related injury and death of cetaceans caused by anthropogenic trauma. The developed technique may also be applied to live cetaceans with abnormal craniocervical relationship to aid diagnosis and guide corrective therapy.

In addition to this quantitative data, adjacent neck structures should also be evaluated for the presence of additional signs, including significant prevertebral soft tissue swelling, cervical spinal cord deficits, respiratory tract collapse and subarachnoid hemorrhage at the AOJ. The presence of large volume of gas between the blubber and muscle layers of neck regions might also indicate the bycatch, off-gassing of supersaturated blood and tissues during or after stranding, sepsis or decomposition^38,39^, which might give clues to an AOD diagnosis.

An AOD may be caused by different traumatic mechanisms, all having in common the transmission of excessive force to the AOJ, leading to widespread ligamentous disruption and deviation from the normal distances between bony structures in AOJ. In cetaceans, a number of ligamentous structures, such as capsular ligament (between occiput and atlas) and central ligaments, are believed to act as important stabilizers of the AOJ^3,40–42^. In the present study, PMCT was capable to recognize cervical osseous landmarks; however, ligamentousstructures cannot be easily visualized in PMCT images^40^. Magnetic resonance (MR) imaging provides precise anatomical information about soft tissue injury. Previous reports in humans have identified disruption of the tectorial membrane, cruciate and alar ligaments and effusion within the AOJ as signs of AOD in MR images^28,43–45^. Further studies on using PMMRI for the detection of morphological changes in these ligamentous structures would be of great practical value in describing any ligamentous injuries that characterized AOD in cetaceans.

It is also important to note that, carcasses with bony abnormality of the cervical spine and cervical spine injury (12 out of 83 carcasses), and carcasses with unfused crania and atlas (16 out of 83 carcasses) were excluded in the present study. These cases should also be taken in count of the possible incidence of AOD and actual frequency of occurrence of antemortem AOD. The AOD may be seen due to antemortem or postmortem fractures of the occipital condyles or atlas^46^, or when there is postmortem decomposition of the supporting soft tissues.

## Methods

### Subjects

A total of 83 finless porpoises, NP, NAS and NAA, in which 23 NP (8 males, 13 females, 2 undetermined sex), 52 NAS (26 males, 24 females, 2 undetermined sex), and 8 NAA (4 males, 4 females) were either stranded or by-caught in Hong Kong waters and Yangtze River, were recruited in the present study. The condition of the carcasses was classified using Smithsonian condition codes^47^ and ranged from code 2 to 4.

### Exclusion criteria

Twelve carcasses (3 NP: 1 male, 1 female, 1 undetermined sex; 9 NAS: 3 males, 6 females) with bony abnormality of the cervical spine and cervical spine injury (e.g. fracture of the occipital area of crania and the atlas) were excluded since the bony landmarks for normal craniocervical relationship could be skewed by adjacent tissue decomposition or disruption from bony injury, leading to measurement failure and error. Sixteen carcasses with unfused crania and atlas (10 NP: 3 males, 7 females; 4 NAS: 1 male, 2 females, 1 undetermined sex; 2 NAA: 1 male, 1 female) were also excluded from the present study since the contours of bony landmarks for normal craniocervical relationship were not visually traced, leading to measurement failure.

### PMCT image acquisition

All PMCT scans of NP (14 whole body scans) were performed with a Toshiba 16-row multislice spiral CT scanner Alexion™ (Toshiba Medical Systems, Japan). The scans were operated at 80 to 120 kV, 60 to 200 mA, and 1 mm slice thickness. Scan field of view (sFOV) was ranged from 15.8 to 53.4 cm. All PMCT scans of NAS and NAA (54 whole body scans) were performed with a 64-row dual source CT scanner SOMATOM Definition™ (Siemens Medical Solutions, Forchheim, Germany). The scans were operated at 120 kV, 15 to 450 mA, and 0.6 to 1 mm slice thickness. The sFOV was ranged from 25.6 to 50.0 cm.

### Image processing and evaluation

Volumetric data was reconstructed and reformed for multiplanar reconstruction using open-source Digital Imaging and Communications in Medicine viewer software (Horos Project, 2015, version 2.1.0; www.horosproject.org). The rendered CT images were analyzed with bone window settings (window width, 1500 HU; window level, 300 HU).

**Figure 3:**
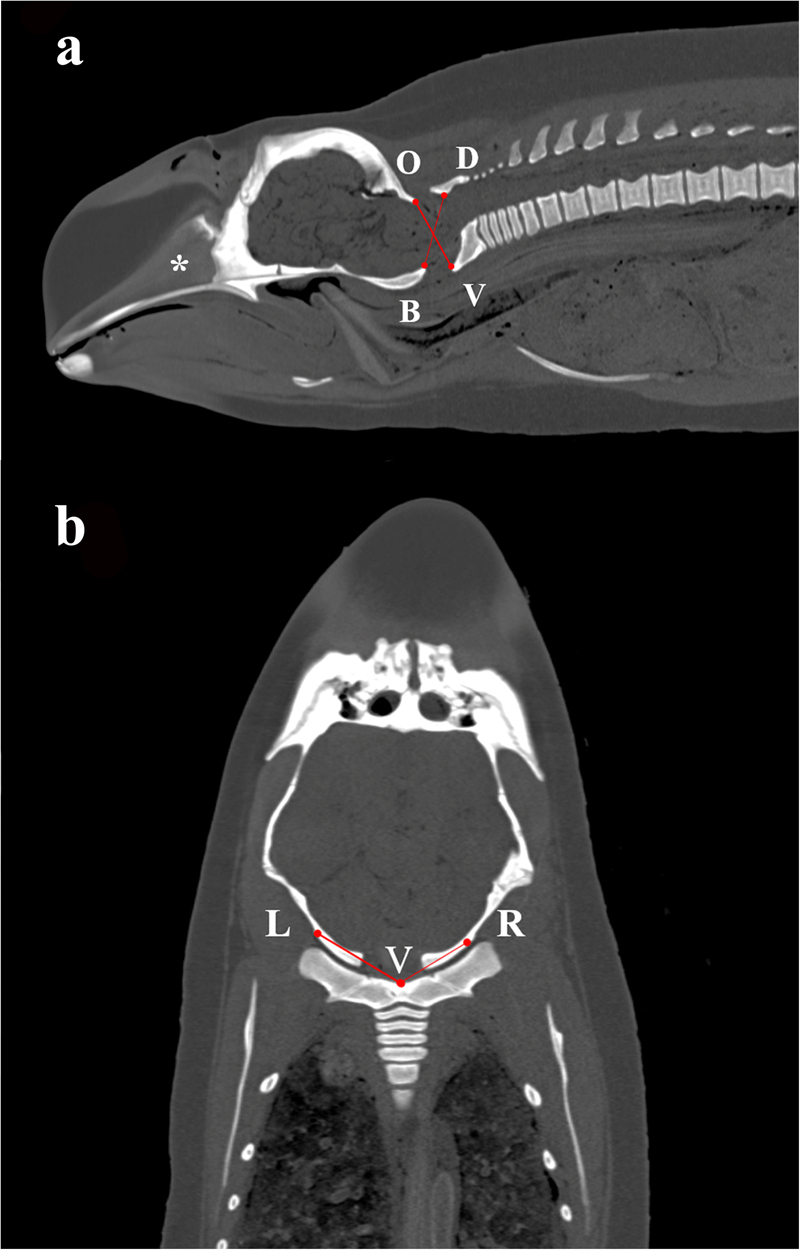
Standardised method of determining craniocervical relationships. (**a**) Indication of basion-dorsal arch (pars basilaris-arcus dorsalis) of atlas (BD) and opisthion-ventral arch (squama occipitalis-arcus ventralis) of atlas (OV) on a reconstructed midsagittal postmortem computed tomography (PMCT) image of the head and neck region, which was chosen as the single image with best alignment of mesorostral groove (*), basion, opisthion, dorsal tubercle of atlas, and ventral tubercle of atlas; (**b**) Craniocervical relationship in medial-lateral dimension was illustrated by the ratio between ventral tubercle of fused cervical vertebral body to right outer margin of occipital condyle (VR) and ventral tubercle of fused cervical vertebral body to left outer margin of occipital condyle (VL), on a reconstructed coronal PMCT image, which was chosen when dorsal most of the fused cervical vertebral body was visualized.

Craniocervical relationship in dorsal-ventral dimension was evaluated by the ratio between BD and OV on a midsagittal PMCT image of the cervical region modified from Powers *et al*.^22^. The reconstructed midsagittal PMCT image was chosen as the single image with best alignment of the mesorostral groove, basion, opisthion and dorsal and ventral tubercle of atlas (Fig. 3a).

Craniocervical relationship in medial-lateral dimension was evaluated by the ratio between VL and VR on a coronal PMCT image. The reconstructed coronal PMCT image was chosen when dorsal most of the fused cervical vertebral body was seen (Fig. 3b).

The complete PMCT data was evaluated, and carcasses with a suspected abnormal craniocervical relationship were identified during PMCT examination by experienced radiological clinician (BK) who had more than 4 year of experience in CT scanning and cetacean virtopsy reporting. In-house veterinarians then performed a detailed conventional necropsy and obtained a complete necropsy report. Antemortem AOD was defined as the presence of subcutaneous edema and hemorrhage in the impact site near the AOJ. The AOD diagnosis was made at a consensus conference attended by the radiological clinician and in-house veterinarians.

All PMCT datasets of the AOJ were also compared with gross anatomical and *in situ* PMCT studies^1,3,48^. Structures not described in previous works were labelled in accordance with the official anatomical terminology^49^.

All procedures in this study were reviewed and approved by the Agriculture, Fisheries and Conservation Department of Hong Kong Special Administrative Region [AF GR CON 09/68 PT.9]. All examinations were performed in accordance with relevant guidelines and regulations.

### Statistical methods

Differences in the craniocervical relationship of dorsal-ventral and medial-lateral dimension between AOD exhibited group and non-AOD exhibited group were analyzed using Kruskal–Wallis test with Dunn’s multiple comparison tests as post-hoc tests (Graphpad InStat 3.05, GraphPad Software San Diego, California). The upper limit of 95% accuracy range was defined as the mean of non-AOD exhibited group + 2 *SD*, while the lower limit of 95% accuracy range was defined as the mean of non-AOD exhibited group − 2 *SD*. For all statistical tests, values of *P* < 0.05 were considered significant.

### Data availability

The data that support the findings of this study are available from the Agriculture, Fisheries and Conservation Department of Hong Kong Special Administrative Region (for NP and NAS) and Institute of Hydrobiology, Chinese Academy of Sciences (for NAA) but restrictions apply to the availability of these data, which were used under license for the current study, and so are not publicly available. Data are however available from the authors upon reasonable request and with permission of the Agriculture, Fisheries and Conservation Department of Hong Kong Special Administrative Region (for NP and NAS) and Institute of Hydrobiology, Chinese Academy of Sciences (for NAA).

## Conclusions

In conclusion, a novel standardised method for determination of normal craniocervical relationships in finless porpoises was first described. The PMCT measurements of normal craniocervical relationship in both dorsal-ventral (mean BD/OV: 0.87 ± 0.24 [2 *SD*]) and medial-lateral dimension (mean VR/VL: 0.98 ± 0.17 [2 *SD*]) was the first reported. The 95% accuracy ranges of the normal craniocervical relationship in dorsal-ventral and medial-lateral dimension were 0.63 to 1.11 and 0.82 to 1.15 respectively. The PMCT measurements together with evaluation of adjacent neck structures could serve as an effective tool for AOD diagnosis.
